# Some Prescriptions with Their Dispensing Difficulties.—II

**Published:** 1909-03-27

**Authors:** 


### Therapeutics and Pharmacy.

### SOME PRESCRIPTIONS WITH THEIR DISPENSING DIFFICULTIES.—II.

How should the following mixture be dispensed?

℞ Ammon. Chlorid. }

Pot. Iod. } ... ... aa ʒij.

Ammon. Carb. ... ... ... ... ʒiss.

Chloral Hyd. ... ... ... ... ʒiv.

Tinct. Lobel. Æth. ... ... ... ʒij.

Tinct. Camph. co. ... ... ... ... ℥j.

Ext. Glycyrrh. liq }

Syr. Simp. } ... ... aa ad ℥iv.

Ft. mist.

Chemical reaction between the chloral hydrate and the ammonium carbonate cannot be prevented here, but it may be hindered to some extent by dissolving the chloral hydrate in the syrup, and adding it at the end to the other ingredients, previously mixed together in a mortar. The prescription is clearly not a good one.

The latter remark applies also to the following:—

℞ Calcii Chlor. ... ... ... ... ... ʒij.

Ext. Glycyrrh. Liq. ... ... ... ℥j.

Aq. ... ... ... ... ... ... ad ℥iij.

Ft. mist.

The result of mixing the above together is a soupy brown opaque fluid; the precipitate might be suspended by the addition of two or three grains of powdered tragacanth to the prescription; but it is far better not to order liquid extract of liquorice and calcium chloride together.

The following is an extreme example of a mixture of incompatibles:—

℞ Pot. Iodid. ... ... ... ... ʒiij.

Sodii Nitritis ... ... ... ... gr. vj.

Acid. Formic. (25 per cent.) ... ... ʒiij.

Aq. Chloroform. ... ... ... ad ℥vj.

Ft. mist.

The formic acid will liberate nitrous acid from the sodium nitrite and hydriodic acid from the potassium iodide. The nitrous and hydriodic acids will then react together, producing free iodine; the latter will be in great part precipitated, but a portion of it will, in the nascent state, oxidise part of the formic acid.

℞ Phenazoni
Quin. Sulph.
Sodii Salicyl. ... ... ... aa gr. xlviij.
Aq. ... ... ... ... ad ℥vj.
Ft. Mist.

The above prescription is difficult to dispense unless considerable forethought is taken. It may be done by dissolving the phenazone and the salicylate in 3 oz. of the water in a measure, shaking up the quinine with the remainder of the water, and then gently mixing the two fluids. A little mucilage should really be included in the prescription also.

In conclusion we would reiterate that the medical student of to-day loses much by the absence from his curriculum of serious and practical dispensing. The loss is felt both by the prescription writer, who does not dispense, and by the doctor who does his own dispensing. The student should be urged to make voluntary experiments in his hospital dispensary. Practice alone can lead to the avoidance of difficulties such as the above.

